# Why do knees after total knee arthroplasty fail in different parts of the world?

**DOI:** 10.1016/j.jor.2020.12.007

**Published:** 2020-12-31

**Authors:** Dominic T. Mathis, Michael T. Hirschmann

**Affiliations:** a University of Basel, CH-4051, Basel, Switzerland; b Department of Orthopaedic Surgery and Traumatology, Kantonsspital Baselland (Bruderholz, Liestal, Laufen), CH-4101, Bruderholz, Switzerland

## Abstract

**Objective:**

The aim of this narrative review was to provide an overview of failure modes after total knee arthroplasty in different parts of the world based on data from worldwide representative studies and National Joint Registries.

**Methods:**

A review of the available literature was performed using the keyword terms “total knee arthroplasty”, “revision”, “failure”, “reasons”, “causes”, “complications”, “epidemiology”, “etiology”; “assessment”, “painful knee”, “registry” and “national” in several combinations. The following databases were assessed: Pubmed (https://pubmed.ncbi.nlm.nih.gov), Cochrane Reviews (https://www.cochrane.org), Google Scholar (https://scholar.google.com). In addition, registry data were obtained directly from national registry archives. Due to the heterogeneity of available data it was decided to present the review in a narrative manner.

**Results:**

Current literature report that infection has become the primary acute cause of TKA failure, while aseptic loosening and instability remain the overall most frequent reasons for revisions. Based on national registries certain tendencies can be deducted. The predominant overall failure mode of aseptic loosening is particularly found in Japan, United Kingdom, New Zealand and Switzerland. Leading early TKA failure mode represents infection with percentages of 20–30% in Sweden, Australia, New Zealand, Japan and the United States. Higher numbers could only be found in clinical studies on the Asian continent such as Korea (38%), China (53%), Iran (44%) and India (87%).

**Conclusion:**

Although there are regional differences in TKA failure modes, TKA fails worldwide especially due to infections and aseptic loosening. It is important to diagnose these in good time and reliably using appropriate, standardized diagnostics in order to recommend the best possible therapy to the patient.

## 1 Introduction

While most patients after total knee arthroplasty (TKA) usually recover well and experience pain relief within 3–6 months,^1^ about 10–30% of the patients report ongoing pain or are not satisfied.^2^ Reasons for dissatisfaction after TKA are recurrent knee pain, limited knee function and the failure to meet preoperative expectations.^3^, ^4^, ^5^, ^6^, ^7^, ^8^ The causes for persistent or recurrent pain after TKA are manifold. Historically, the most common failure mode besides periprosthetic joint infection includes aseptic loosening, instability and malalignment. Along with improved polyethylene quality polyethylene wear, subsequent osteolysis and loosening have become less prevalent.^9^, ^10^, ^11^ Recent studies reported that periprosthetic joint infections have become the primary *acute* cause of failure, while loosening and instability remain the *overall* most frequent reasons for revisions.^12^, ^13^, ^14^ The consequences for failed TKA become greater with the increasing number of interventions. The demand for TKA in the United States is projected to rise by 673% by 2030, and the revision TKA by 601%.^15^ An ageing population, a rising prevalence of risk factors such as obesity, and the necessity to maintain active lifestyles will continue to feed this trend in developed countries.^15^^,^^16^ A consequential rise in failed TKAs is inevitable, and they present a significant financial and health-related burden to patients and healthcare systems.^17^^,^^18^ However, the distribution and frequencies of failure modes vary according to type of study, registries, nationality and cohort.

The understanding of failure in TKA is a pertinent one. Only if we understand why a TKA fails one can tailor the diagnostic and treatment algorithm in this direction. In the current climate of increasingly short resources an efficient diagnostic and treatment algorithm should be able to detect the most important failure modes in TKA.

Although it could be speculated that there are wide regional and ethnical differences for failure modes in TKA, to date there is no specific literature presenting this information in a comprehensive manner. The pertinent question is whether the failure modes in TKA differ with regards to contents or countries respectively.

Hence, it is the purpose of this narrative review to provide an overview of failure modes after TKA in different countries based on data from worldwide representative studies and National Joint Registries.

## 2 Methods

A systematic review of the available literature was performed using the keyword terms “total knee arthroplasty”, “revision”, “failure”, “reasons”, “causes”, “complications”, “epidemiology”, “etiology”; “assessment”, “painfull knee”, “registry”, “national” in several combinations. The following databases were assessed: Pubmed (https://pubmed.ncbi.nlm.nih.gov), Cochrane Reviews (https://www.cochrane.org), Google Scholar (https://scholar.google.com/).

All the publications from January 01, 2000 to July 01, 2020 were searched. The search was limited to English and German studies only (Table 1). Studies in other languages were not included in this review. In addition, registry data were obtained directly from national registry archives (Table 2).

**Table 1:** Clinical studies by failure modes (%). Sharkey et al.,^13^^,^^22^ Hossain et al.,^10^ Le et al.,^23^ Haasper et al.^27^ and Khan et al.^45^: First number is early (<2years) failures, second number is late failures; Schroer et al.^11^: First number is early (<2years) failures, second is overall failures. Dalury et al.^26^ table: First number is early (<5years) failures, second is late (**≥**5 years) failures. *The authors grouped stiffness/pain under one category.

Ref. and publication year	Origin	N	Aseptic Loosening	Wear; lysis	Instability	Infection	Arthro-fibrosis	Patello-femoral or extensor mechanism^a^	Peri-prosthetic fracture	Mal-alignment	Pain	other^b^
Fehring et al.,^12^ 2001	US	279	3	7	**26**	**38**	–	8	–	12	–	**18**
Sharkey et al.,^13^ 2002	US	212	17/**34**	12/**44**	21/22	**25**/7.8	17/12	10/2	2.8/2.8	12/12	–	–
Sharkey et al.,^22^ 2014	US	781	**22.8/51.4**	2/4	**6.1/10.3**	**37.6/22**	–	3.5	–	3/2	–	–
Mulhall et al.,^14^ 2006	US	318	**41**	**24.9; 59.4**	**29**	10.4	–	1	–	9	–	8
Schroer et al.,^11^ 2013	US	844	**19/31**	1/10	**25/19**	**23/16**	13/7	7/6	2/3	8/7	–	2/1
Delanois et al.,^31^ 2017	US	337597	**20.3**	2.6	7.5	**20.4**	–	–	–	–	–	**12**
Bozic et al.,^28^ 2010	US	60436	**16**	5/3	–	**25**	–	–	2	–	–	**32**
Le et al.,^23^ 2014	US	253	14/13	2/9	**26**/18	**24/25**	18/14	4	2	7	2	**30/34**
Dalury et al.,^26^ 2013	US	820	14.8/**24.1**	0.2/**34.1**; 1.3/8	24/15	**30**/8	14.5/6.6*	–	1.8/1.3	3/1.3	14.5/6.6*	3.1/1
Abdel et al.,^39^ 2017	US	112	**13**	7	**16**	**54**	1	–	8	–	–	–
Pitta el al,^40^ 2018	US	405	**21.2**	2.5	**24.4**	**25.7**	14.1	3	3.5	2.5	1.3	2
Haasper et al.,^27^ 2012	GER	150	5/**25**	0/3	9/**16**	10/6	6/**11**	–	–	2/3	1/1	–
Postler et al.,^41^ 2018	GER	402	**21.6**	5.2	6.7	**36.3**	4.5	3.7	**13.7**	–	6	8
Thiele et al.,^42^ 2015	GER	358	**21.8**	7	**21.8**	14.5	4.5	7	3.3	**20.7**	–	–
Hossain et al.,^10^ 2010	UK	349	3/**12**	1/**12**	4/3	**12/21**	2/1	3/2	2/7	4/3	–	9
van Kempen et al.,^43^ 2013	NL	150	**27**	–	15	**23**	10	–	–	**25**	–	–
Kasahara et al.,^25^ 2013	Japan	147	**40**	9	9	**24**	–	–	4	–	–	**14**
Koh et al.,^24^ 2014	Korea	634	**33**	**13**	7	**38**	3	1	2	1	1	8
Huang et al.,^44^ 2015	China	181	**16**	4.4	6.6	**53**	**10**	5	2.8	–	–	2.2
Khan et al.,^45^ 2017	India	53	2.5/**43**	–	–	**87/35**	–	2	**6**	2	–	4
Motififard et al.,^46^ 2015	Iran	36	**25**	–	2.8	**44.4**	–	**25**	2.8	–	–	–

The three most common reasons are in **bold**.

N, qualified for revision surgery. US, United States; UK, United Kingdom; GER, Germany; NL, Netherlands.

a Includes patellar dislocation, resurfacing due to progression of osteoarthritis, erosion, failure, and patellar pain.

b Includes incorrect sizing, metal sensitivity, implant breakage, and other or missing reasons.

**Table 2:** Registries by failure modes (%). N, qualified for revision surgery. *First number is overall revision percentage, second number is early revision (<3 months from primary procedure) percentage.

Registry	N	Aseptic Loosening	Wear; lysis	Instability	Infection	Arthro-fibrosis, stiffness	Patello-femoral or extensor mechanism^a^	Peri-prosthetic fracture	Mal-alignment	Pain	other^b^
Norway^38^	8147	**20**	2.8	14.9	**16.2**	–	3.8	3	6.7	**24.7**	–
Sweden^37^	4685	**28**	5	**15**	**30**	–	13	3	–	–	6
Germany^47^	13378	**25**	5.7; 1.1	8.9	**14.7**	4	4.2	3	1.8	–	**31.6**
Switzerland^48^	8340	**34.5**	5.8	15.9	**17**	4.9	**25.7**	2.8	10.4	12.7	11.7
United Kingdom^49^	60294	**37**	12.9	**15.9**	**15.7**	5.4	12.1	3.8	6.9	14.7	14.6
Australia^50^	24722	**25**	3.9	8.1	**23.3**	3.6	**15.5**	3	2.2	8.2	7.3
New Zealand^51^	3652	**35.9**	–	–	**26.6**	–	25.1	3	–	**29.4**	–
United States^52^	49491	**25**/2.8*	2.9/0.3*	12.6/6.7*	20.5/63*	–	–	2.3/3.9*	–	–	37/23*
Japan^53^	1679	**44.4**	12	13.5	**32**	3	–	2.2	–	–	**16.8**

The three most common reasons are in **bold**.

a Includes patellar dislocation, resurfacing due to progression of osteoarthritis, erosion, failure, and patellar pain.

b Includes incorrect sizing, metal sensitivity, implant breakage, and other or missing reasons.

All peer-reviewed articles were considered. Randomized controlled trials (RCTs), prospective trials and retrospective studies as well as reviews and case reports were included in this review.

Due to the heterogeneity of available data it was decided to present the review in a narrative manner. One author extracted data from all the selected original articles and registries, which was repeated by the other author. If there was no agreement between the two, the senior author was consulted. Data were extracted from each included article and entered into a spreadsheet for analysis. Pertinent information extracted included author, date, origin of the study, N and failure modes.

## 3 Results

### 3.1 Registry data

The first nationwide arthroplasty registries were developed in the 1970s in Sweden. The Swedish Knee Arthroplasty Register was started in 1975.^19^ Many more countries have developed arthroplasty registries since then: Australia, Belgium, Canada, Croatia, Denmark, Netherlands, Egypt, Finland, France, Germany, Hungary, New Zealand, Japan, Norway, Pakistan, Portugal, Romania, Slovenia, Slovakia, Switzerland, and the United States. The National Joint Registry of England, Wales, Northern Ireland, and the Isle of Man covers most of the United Kingdom but does not include Scotland (it has its own registry). In Italy, there is the Registro dell'implantologia Protesica Ortopedica (R.I.P.O) and in Spain, there is the Catalan Arthroplasty Register. The United States has national, regional, and institutional arthroplasty registries. Most recently in 2016, Iran introduced the National Iranian Joint Registry, however first results have not been published yet.^20^ South Korea employs national medical data collected by the Health Insurance Review Agency (HIRA). The HIRA database contains reimbursement records from all medical facilities of all citizens including demographic data and surgery details (e.g. TKA).^21^ However, there are considerable linguistic difficulties in the analysis of these records.

### 3.2 Longitudinal studies

The spread of countries in which longitudinal studies have been performed is quite similar to the national registries. The most represented countries are the United States, United Kingdom, Germany, Korea and Japan.^10^, ^11^, ^12^, ^13^, ^14^^,^^22^, ^23^, ^24^, ^25^, ^26^, ^27^, ^28^, ^29^, ^30^, ^31^ African, South American, Asia-Pacific countries are clearly underrepresented in literature and therefore the figures given worldwide are not based on standardized, globally, collected data.

### 3.3 Defining knee arthroplasty failure

The results of arthroplasty have traditionally been reported using survivorship analysis, with implant revision as the primary endpoint.^32^ Given that the primary indication for TKA is severe pain, it has been suggested that pain (or even functional deficiency), represents a more appropriate outcome measure for treatment success following TKA.^33^ Function, performance and activity levels are also accepted as valid markers of treatment success and may be more appropriate for the younger population.^34^ The use of revision alone to indicate success or failure of a treatment such as TKA may well introduce bias in favour of the success. Namely, patients who are unable or unwilling to undergo revision, would fail on the basis of a different classification system (e.g. pain). Price et al. highlighted this problem reporting a minimal 12-year cumulative survival of 82.2% using revision as an end point. This decreased dramatically to 59% if moderate or severe pain was included as an end point.^18^ This shows, that the use of different outcome measures substantially alters the proportion of TKAs which are deemed successful. Patient Reported Outcome Measures (PROMs) are becoming popular in the assessment of satisfaction and health-gain postoperatively. However, many PROMs have been developed to measure quality of life and functional outcomes, although not all of them have optimal measurement properties.^35^ Thus, when choosing an instrument to monitor TKA outcomes, consider both the quality of the instrument and the constructs it proposes to assess.

Moreover, the definition of revision may raise further questions: Implant revision remains the benchmark outcome by which survival of orthopaedic implants is assessed,^36^ although it is affected by a number of factors (e.g. health status, inadequate implant monitoring, missed diagnosis of implant failure). The term “revision” must, however, be clearly defined. For example, the Swedish registry uses the following stringent definition: any new surgical procedure during which one or more prosthesis components are replaced, removed, or added.^37^ The Norwegian registry, in contrast, defines revision as the removal of all the implant components and therefore classifies patellar resurfacing, for instance, as a simple re-operation.^38^

### 3.4 Global overview on failure modes

The studies and registries in Table 1, Table 2 show that aseptic loosening, instability and infection represent the three major overall causes for TKA revisions.^11^^,^^22^^,^^37^^,^^39^^,^^40^^,^^49^ Moreover, Table 1 highlights the importance of wear/osteolysis as etiology of TKA failures.^10^^,^^13^^,^^14^^,^^24^^,^^26^ However, modes of failure from polyethylene wear and subsequent osteolysis became less prevalent as a result of improved polyethylene quality and manufacturing, better seating and anchorage mechanisms, more highly polished surfaces, improved surface morphology that avoids point-to-point contact, and sterilisation of polyethylene in oxygen-free environments.^11^^,^^24^^,^^25^^,^^28^ This led to a shift of failure modes over time. Whereas in the very beginning of the 21st century Sharkey et al.^13^ and Mulhall et al.^14^ identified polyethylene wear as the most common etiology for TKA failure, nearly 20 years later a shift towards aseptic loosening and infection was to observe according to recent studies.^31^^,^^40^^,^^41^^,^^44^^,^^45^

In 2014 Sharkey et al. provided a 10-year update on their experience of performing 781 revisions of 10′003 total procedures (7.8% revision rate).^22^ They found a dramatic decrease from 25% to 3.5% in the rate of polyethylene wear as the cause of revision. Early (<2 years) failures with 37.6% infection were most common, and more than half (51.4%) of the 62.4% late (>2 years) revisions were aseptic loosening. This observation can be confirmed by current national arthroplasty registries (Table 2). Wear/osteolysis is no longer accounting for the majority of TKA failures (<13%), but currently aseptic loosening (25–44%) with a maximum value in Japan with 44%.^53^ Not surprisingly, with its early failure numbers, the American registry points to the leading role of infection in this time spectrum (<3 months from primary procedure = 63%).^52^ Also most clinical studies, which distinguished between early and late failure modes, reported infection as the most common reason for early failures.^10^^,^^11^^,^^13^^,^^22^^,^^23^^,^^26^^,^^27^^,^^45^ The registries of New Zealand, Norway and England, Wales, Northern Ireland and the Isle of Man reported high rates of revision due to unexplained pain, with an incidence as high as 29.4% observed in New Zealand knee revisions.^38^^,^^49^^,^^51^ The National Joint Registry of Switzerland reported the highest incidence of revision due to patello-femoral or extensor mechanism failure (25.7%) and malalignment (10.4%).^48^ Only New Zealand showed similarly high patello-femoral or extensor mechanism failure rates (25.1%).^51^ All included national registries showed similar frequencies of instability, arthrofibrosis/stiffness and periprosthetic fractures (Table 2).

In recent years, there has been an increase in patients reporting symptoms relating to knee instability in the United States and Australia: both registries have reported rising rates of revision for knee instability over the last decade. Whilst in 2008 the Australian knee arthroplasty registry identified instability as the ninth most common reason for all revisions at 2.9%,^54^ the latest data of 2019 showed an increase to the fifth most common reason for revision at 8.1%.^50^ Similarly, a doubling of the percentage of revision interventions due to instability was observed in the United States from 5.9% to 12.6% between 2014^55^ and 2019.^52^ Long-term studies, which were carried out in the United States over the last 20 years, could also confirm this trend from the register data (Table 1). However, this shift in the causes of revision does not apply to Europe.^41^^,^^42^^,^^56^, ^57^, ^58^, ^59^ It is unclear whether this trend is due to an increasing incidence in these countries, or an increased awareness of the diagnosis. Against this background, it is crucial to point out the various failure modes and take a critical look at their pathogenesis.

### 3.5 Defining failure modes

#### 3.5.1 Instability

Instability can arise from component loosening, component breakage, polyethylene wear, ligamentous instability or surgical error in relation to implant size or balancing of the knee.^60^ Thus, it can often be attributable to surgical technique and is mostly preventable as it relates to poor ligamentous balancing, and unequal flexion and extension gaps. Surgeons must understand the delicate balance of stability in both the coronal and sagittal planes. Within all current TKA implant systems, there exists a continuum of constraint for each individual patient. It seems, however, that every knee has its own “envelope of alignment”, in which the knee can function without limitations. The question of which ‘envelope of alignment’ fits which individual patient is currently subject of research.^61^

Discussion around clinical cases and presentations on orthopaedic congresses has suggested that there is a growing awareness of instability as a mode of implant failure amongst surgeons. A number of definitions of knee instability exist in literature.^62^ Instability may refer to the whole knee or may be used interchangeably with the term loosening, which more appropriately refers to a specific component and its fixation to the bone. In addition, symptoms that present and appear to be caused by instability may also be due to a number of other factors including patellofemoral articulation, muscular weakness, component loosening, and infection.^63^

In a systematic review including 22 studies, Wilson et al. analysed in 2017 the pathology of knee instability as the primary cause of failure following TKA.^62^ They reported a time to failure between primary and revision TKA of 44.7 months and a mean age at time of revision of 67.6 years. A gender distribution was identified with approximately 16.4% more females revised for instability. Further they noted, that a mere three studies described the type of instability. These results are consistent with Australian national data, which suggests revision rates are higher in patients who are less than 70 years of age when the primary knee surgery was performed. Furthermore, more than a third (35.7%) of all knee arthroplasty revisions reported by the Australian National Registry occurred in the 65 to 74 age bracket.^50^

With instability increasing as a cause of revision TKA, a clear understanding of the factors contributing to instability and subsequent revision is imperative. An assessment of an unstable knee has been recently described by Petrie et al.^64^ and Cottino et al.^65^ The use of clinical and radiological assessment including stress radiographs is considered in these papers to obtain the correct diagnosis. A decisive diagnostic factor is the clinical history. Patients with symptomatic instability, particularly in flexion, report a common series of symptoms including a feeling of insecurity in the knee without frank giving way, difficulty with stairs, recurrent knee swelling and anterior knee pain.^65^ Therefore, a combination of both assessments is required to accurately confirm the diagnosis of instability and to exclude other diagnoses, which may elicit a different treatment approach.

#### 3.5.2 Infection

Periprosthetic joint infection (PJI) is one of the most challenging and frequent complication after lower-extremity joint (hip and knee) arthroplasty. Delanois et al. reported in 2017 that infection was the most common etiology for revision of total knee arthroplasty (20.4%) in the United States.^31^ The American registry even tops this number by 63% infections accounting for early failures (<3 months from primary procedure).^52^ Studies from Korea, China and India report infection rates of 38%, 53% and 87%, respectively.^24^^,^^44^^,^^45^

PJI can be divided into acute and delated onset. The actual numbers are a matter of debate. Whereas acute infection usually can be diagnosed easily, low-grade PJI diagnostics remain a considerable hurdle.^66^^,^^67^

Standardized definitions by the Musculoskeletal Infection Society (MSIS) and the Infection Disease Society (IDSA) are widely accepted by researchers and surgeons.^68^^,^^69^ However, through the years various definitions have been proposed and yet there is no agreement on the definition of PJI that has been reached. New diagnostic criteria are proposed constantly for diagnosing PJI more accurately.^68^^,^^70^^,^^71^ The criteria were last adjusted in 2018 at the International Consensus Meeting on Musculoskeletal Infection in Philadelphia.^72^

Acute hematogenous or postoperative infections are caused by aggressive, high-virulence bacteria and usually meet the above mentioned standardized diagnostic criteria. Conversely, intermediate or chronic infections are predominantly caused by slow-growing, low-virulence pathogens (e.g. Propionibacterium acnes and Staphylococcus epidermidis).^73^ In these patients not only the clinical signs of infection are missing, but synovial fluid and intraoperative tissue cultures are showing false negative rates up to 53%.^74^^,^^75^ Therefore, those cases are misdiagnosed as aseptic loosening or stiff-knee. Subsequent insufficient revision surgery results in soft-tissue damage and bone loss, early failure, and additional revision procedures. Consequently, identification of low-virulence pathogens is the current challenge in PJI diagnostics.^76^

#### 3.5.3 Arthrofibrosis/stiffness

Arthrofibrosis is a debilitating complication after knee surgery that may also result in pain that does not subside at predictable time points, pain with palpation, deficiency of patellar movement with quadriceps muscle contraction, or a knee joint that is warm or swollen unrelated to effusion. There is a lack of consensus on the diagnostic criteria for arthrofibrosis of the knee, which obscures its true prevalence after surgical procedures.^77^ The etiology of arthrofibrosis is multifactorial, and numerous preoperative, intraoperative, and postoperative risk factors have been identified.^78^ It has many patient-related factors including diabetes, rheumatoid arthritis, ankylosing spondylitis, and restricted pre-operative range of movement.

After TKA, arthrofibrosis shows a reported incidence of 1%–13% postoperatively.^78^ The range of values can be attributed to the varying quantitative thresholds of flexion and/or extension loss used to define arthrofibrosis, as described above. As a predominant failure mode, arthrofibrosis accounts for 28% of hospital readmissions due to surgical complications within 90 days of discharge, and 10–15% of all revisions within 5 years of initial surgery within the United States.^13^^,^^23^^,^^26^^,^^40^ For Europe, both registry and study data show lower rates at around 5%.^10^^,^^41^^,^^42^^,^^47^, ^48^, ^49^ The lowest numbers were published in Korea, Japan and Australia with 3% failure due to arthrofibrosis (Table 1, Table 2).^24^^,^^50^^,^^53^ Hence, you could speculate that in countries with shorter mean length of hospital stay (LOHS) after TKA, such as the United States with a mean LOHS of 3.5 days^79^ show higher arthrofibrosis complication rates than countries with longer LOHS (e.g. Japan 32 days^80^). The postsurgical rehabilitation process with accelerated and constant physiotherapy might be more efficient during hospital stay compared to ambulatory treatment where the patients are left to themselves and thus arthrofibrosis might manifest.

Unfortunately, pain and function scores after revision TKA for arthrofibrosis have been shown to still lag behind scores for revision TKA for other reasons.^81^ Arthrofibrosis may still occur after a revision due to the surgical trauma, postsurgical rehabilitation process, or patient predisposition, as those patients who require a revision due to arthrofibrosis are also those with the highest risk for the persistent development of severe fibrosis. Kim et al. found that over 25% of revision TKAs due to stiffness required a second revision.^82^

#### 3.5.4 Aseptic loosening

Unlike other etiologies that occurred either early or late, aseptic loosening occurred frequently throughout follow-up. Although extensively studied, this failure mode is not particularly well understood, and certainly is the “catch-all” diagnosis of failed knees in which an alternative diagnosis could not be made. This category represents an extended list of subcategories of failure modes. Fehring et al. reported 13% of early failures were failures of cementless fixation, pointing out that the etiologies of aseptic loosening of cementless implants will vary from cemented implants.^12^ In addition, variations in surgical technique among surgeons may contribute to aseptic loosening. Higher loosening has been demonstrated in cemented knees when the tibial stem is left uncemented.^83^ However, latest results from a prospective, randomized trial did not show any radiographic evidence of component subsidence or loosening neither in the cemented nor in the uncemented TKA cohort (two years postoperatively).^84^ Variations among implants within a single product line and among different manufacturers may lead to different potential failure modes. Tibial trays with short stems have been associated with an increased rate of aseptic loosening.^85^ Implant specific failure reports have shown that specific implants have had a high early rate of aseptic loosening.^86^

The authors of this review would suggest, that efforts need to be made to better define and address aseptic loosening. The term aseptic loosening should be used when the implant was initially well fixed and subsequently loosened. These patients were generally well satisfied with their knee initially. This would contrast with “failure of fixation” for implants that were never secure in which patients were never satisfied with their knee.

In literature different diagnostic values are reported for the various imaging modalities.^87^ One of the highest sensitivities and specificities for detection of tibial or femoral component loosening of all imaging modalities has been shown for combined single-photon emission computerized tomography and conventional computer tomography (SPECT/CT).^88^ Therefore, it can be speculated that the inter-regional comparability of aseptic loosening is lacking due to the different imaging techniques in the aforementioned studies and registries (Table 1, Table 2).

Nevertheless, it should not be left un-attempted to deduce explanations for increased aseptic loosening rates in Asian countries (Japan 40%,^25^^,^^53^ India 43%,^18^ Korea 33%^24^). Kim et al. investigated the anthropometric differences of Koreans from Westerners. They found shorter stature, less weight, and smaller skeletal structure and a higher incidence of constitutional varus alignment of the lower extremity in Koreans.^89^ Culturally, Koreans and other Asian populations have life styles that demand high flexion positions of the knee such as squatting, kneeling, and cross-legged sitting. High-flexion positions after TKA and engagement of seniors in frequent social and leisure activities are thought to be linked with the growing incidence of aseptic loosening.^90^ Cho et al. reported that early aseptic loosening of the femoral component did not occur in patients who had been prohibited from engaging in squatting and kneeling after high-flexion TKA.^90^ This thesis is supported by the results of Motififard et al., who described aseptic loosening as the second most frequent cause of TKA failure among the Iranian population with a Muslim proportion of 99.5% and kneeling prayers several times a day.^46^

#### 3.5.5 Malalignment

Malalignment has been a great subject of debate for a long time and still is. However, most surgeons are in agreement that correct limb alignment at the time of primary TKA is essential to a well-functioning, durable implant. This postulation has been further supported by the results of this study group in 2016.^91^ Hirschmann et al. compared symptomatic with asymptomatic patients after primary TKA using SPECT/CT for the assessment of TKA component position and bone tracer uptake patterns. Symptomatic knees after TKA showed significantly less optimal positioned TKA than asymptomatic ones. The study group further demonstrated that 3D-CT is the most accurate method to determine TKA component position relating to the mechanical axis. However, most authors use axial 2D-CT slices for measurement of component position, although it has been shown that these measurements are very variable and less reliable.^92^ It is therefore not surprising that countries with highly specialized medical care systems (Switzerland,^48^ The Netherlands,^43^ Germany^42^) and the routine use of 3D-CT scans show higher malalignment rates than countries without these possibilities (Table 1, Table 2).

#### 3.5.6 Pain

Approximately 20% of the patients after primary TKA report ongoing or recurrent pain or an unsatisfying outcome - the causes for that are manifold.^2^ All aforementioned failure modes but also non-knee joint related causes (e.g. vascular pathologies, psychological disorders, back pain or hip problems) may apply.^6^ The diagnostic process is demanding as it requires a detailed patient history, a thorough clinical examination, radiological, serological and microbiological investigations.^6^^,^^8^ Only the integration of all these factors into the diagnosis allows the failure mode of the TKA to be found reliably. Therefore, a standardized diagnostic algorithm for painful patients after TKA in the setting of a specialized TKA centre is inevitable.^91^

The authors agree with the common sense, that revision operations should only be performed if the cause(s) of the complaints described have been identified and fit the clinical picture, as revision surgery for unexplained pain has consistently been shown to result in poor outcomes.^6^^,^^7^^,^^93^, ^94^, ^95^ Thus, the diagnosis pain should not be an indication for a TKA revision but rather the symptom that has an underlying cause. With this considered, Sharkey et al.^13^ for instance, but also most other clinical study groups (Table 1) excluded all patients with unidentifiable causes of pain from their series and did not offer knee revision as an option.

The fact that this failure mode is prevalent in the registries of New Zealand^51^ and Norway^38^ (Table 2) suggests that either the underlying pathology (=failure mode) has not been registered as primary diagnosis or that revision surgery has been performed without detailed and differentiated diagnostic work-up.

#### 3.5.7 Patellofemoral or extensor mechanism problems

This failure mode represents a very heterogeneous group. It includes patellar dislocation, resurfacing due to progression of osteoarthritis, erosion, failure, and patellar pain. A large proportion of this pathology constitutes the subgroup “secondary patella resurfacing”. This, in turn, depends on the proportion of primary patella resurfacing during primary TKA. Registry data demonstrate, that countries with high rates of patellofemoral or extensor mechanism problems (Switzerland 25.7%,^48^ Australia 15.5%^50^) also show high proportions of secondary patella resurfacing revisions (Switzerland 21.9% (including conversion from unicompartmental to total knee arthroplasty), Australia 19.8%). On contrary, countries like Germany and Norway with low rates of patellofemoral or extensor mechanism problems (Germany 4.2%,^47^ Norway 3%^38^) show lower secondary patella resurfacing proportions (Germany 8.5%, Norway 12.5%).

There are also certain culturally determined peculiarities in this failure mode. Iran, which is characterized by a very high Muslim proportion and kneeling prayers several times a day, ranks patellar complications (25%) as second most common TKA revision reason (together with aseptic loosening), right after infection (44.4%).^46^

## 4 Discussion

In this review of literature, we analysed TKA failure modes in different parts of the world. Regional discrepancies were found, however, the critical examination of these various failure modes clearly highlights the limitations of an international comparison.

Firstly, it becomes unmistakably obvious that the definition of the failure mode applied in the study or registry is very decisive for the comparison with other results. This also involves the diagnostic instruments used for the assessment of a pathology. For example, Thiele et al. described in detail which criteria have to be fulfilled in order to be assigned to the instability group. In 21.8% of the cases they found instability accounting for the indication for revision.^42^ Conversely, Postler et al. only noted that a “positive history and suitable physical examination” were necessary for the diagnosis of instability. Hence, they only identified instability in 6.7% as failure mode.^41^

Secondly, there are inconsistencies in the categorization of failure modes amongst the registries and studies. In 2015, Niinimäki combined five worldwide registries: Australia, New Zealand, Norway, Sweden, and England and Wales. He pointed out these inconsistencies which cloud the ability to interpret the results.^96^ For example, pain and malalignment are not categories in the Swedish, Japanese and United States registries^37^^,^^52^^,^^53^ whereas wear/osteolysis, instability and arthrofibrosis/stiffness are not being mentioned in the New Zealand registry.^51^ However, the United States registry accounts “other mechanical complications” for 22.5% of TKA revision indications without further defining this category.^52^ Similarly, the German registry coded >30% of the failure modes for “other reasons”.^47^ Niinimäki therefore suggested that standardizing the registries can help in compiling data to draw more compelling conclusions.

Thirdly, the exclusion criteria in clinical studies can be chosen very individually and thus make the comparability of the different studies considerably more difficult. Hossain et al. for instance, they have excluded patients with simple debridement and washout, simple polyethylene exchange, fracture fixation, or an isolated patellar resurfacing from their retrospective review.^10^ On the contrary, Pitta et al. excluded only patients who underwent isolated irrigation and debridement for hematoma without removal of components; isolated polyethylene exchange and patellar resurfacing, however, were not specifically excluded.^40^

Fourthly, when interpreting these TKA failure modes it is important to recognize the strong regional publication bias. The vast majority of all data on this topic were obtained from United States or European patient cohorts. Especially African and South American countries represent a “black box” where no data can be found in literature. Also, on the Arabic and Asian region there are scarce data available in the common electronic libraries. Consequently, the current state of knowledge is built on published data from highly developed countries, and does by far not represent all ethnicities.

## 5 Conclusion

The survivorship of TKAs is determined by the changing demographics of patients, surgical technique and implant-related factors. Newer longitudinal studies and national registries report that infection has become the primary acute cause of failure, while loosening and instability remain the overall most frequent reasons for revisions. Based on national registries certain tendencies can be deducted. The predominant overall failure mode aseptic loosening is particularly found in Japan, United Kingdom, New Zealand and Switzerland. Leading early TKA failure mode represents infection with percentages of 20–30% in Sweden, Australia, New Zealand, Japan and the United States. Higher numbers could only be shown in clinical studies on the Asian continent such as Korea (38%), China (53%), Iran (44%) and India (87%). This implies, that Asian countries are more in the early revision/infection window. Polyethylene wear has been reduced and was not a major cause of failure. Unexplained knee pain after TKA represents in Norway and New Zealand one of the major reasons for revision surgery. Patellofemoral or extensor mechanism problems include many sub-pathologies and is a predominant failure mode in Switzerland, New Zealand and Iran. Malalignment can only be invoked as a reason for TKA revision if the positioning of the TKA components can be determined reliably and accurately. This requires appropriate imaging equipment, which is not available in most parts of the world. Swiss registry data suggest revision rates due to malalignment in 10.4%. For instability, arthrofibrosis/stiffness and periprosthetic fractures no regional patterns can be demonstrated.

To summarize, although there are regional discrepancies of TKA failure modes, TKA fails worldwide especially due to infections and aseptic loosening. It is important to diagnose these in good time and reliably using appropriate, standardized diagnostics in order to recommend the best possible therapy to the patient.

## Declaration of competing interest

No conflicts of interest.
